# *In Vitro* Aging and Fracture Tests on Differently Veneered Partially Stabilized Zirconia Anterior Crowns

**DOI:** 10.1155/2024/2037792

**Published:** 2024-10-09

**Authors:** Andreas Zenthöfer, Ali Ilani, Stefan Rues

**Affiliations:** Department of Prosthodontics, University Clinic Heidelberg, University of Heidelberg, Heidelberg, Germany

**Keywords:** fracture resistance, translucency, veneering, zirconia

## Abstract

**Objectives:** To evaluate the fracture resistance of veneered incisor crowns made from highly translucent zirconia frameworks.

**Materials and Methods:** Ninety-six all-ceramic single crowns were based on either a coping with minimum wall thickness or a cutback framework fabricated from highly translucent zirconia (5Y-PSZ). Each one-third of the specimens was finalized with different veneering ceramics using standardizations and glaze firing. Crowns were luted to cobalt–chromium dies with MDP-containing composite cement. Half of the entire sample underwent artificial aging (chewing simulation and thermocycling) before fracture load tests were conducted using a 6 mm steel sphere applied in a 90° angle to the oral aspect of the crowns with 1.3 mm distance to the incisal edge. Besides descriptive presentation of recorded forces at first damage (F_1d_) and fracture (F_u_), Kruskal Wallis and Mann–Whitney *U* tests were used to analyze data at *α* = 0.05.

**Results:** Directly after manufacturing, incisor crowns of all test groups showed sufficient mean fracture resistances. After artificial aging, crack formation was observed in a high percentage for fully veneered crowns of all test groups, but only for one veneering ceramics with cutback crowns. Mean test forces of unaged crowns were F_1d,mean_ ≥ 422 N | F_u,mean_ ≥ 749 N (fully veneered) and F_1d,mean_ ≥ 644 N | F_u,mean_ ≥ 706 N (cutback) dropped significantly to F_1d,mean_ ≥ 131 N | F_u,mean_ ≥ 223 N (fully veneered) and F_1d,mean_ ≥ 324 N | F_u,mean_ ≥ 524 N (cutback) .

**Conclusions:** Within the limitations of this laboratory study, 5Y-PSZ based anterior crowns can be a viable treatment option. Framework design, choice of the veneering ceramics and artificial aging show relevant effects on the fracture resistances. Concerted veneering ceramics should be used and partially veneering of the zirconia frameworks should be favored over full veneers.

## 1. Introduction

In restorative dentistry the demand of tooth-colored metal free restorations especially in the so-called esthetic anterior zone is more and more increasing [1]. With respect to material evolution zirconia-based restorations have become state of the art for a variety of indications [1, 2]. The automated processing of the CAD/CAM approach, cost effectiveness in comparison to gold-based restorations and good biocompatibility made this material class very competitive [3, 4]. However, a major drawback in veneered all-ceramic restorations is that the most common complication is crack formation within or fracture of the ceramic veneering (adhesive or cohesive fractures; chippings) [5].

In the last years, the esthetics of the classic tetragonal zirconia polycrystal doted with 3 mol% yttria, so-called 3Y-TZP, could be improved by reducing alumina content and raising the sintering temperature. Due to this development and precolored zirconia variants, monolithic zirconia restorations with acceptable esthetics for the posterior region [6] became viable and the use of veneering ceramics could be reduced for some indications. In order to further improve its esthetics, zirconia was modified by adding more yttria (4–6 mol%) resulting in a mix of cubic and tetragonal crystals. When doted with 5 mol% yttria, this partially stabilized zirconia (5Y-PSZ), consists of about 50% tetragonal and 50% cubic crystals. Since with cubic zirconia crystals there is, in contrast to tetragonal zirconia, no transformation toughening, the bending strength of 5Y-PSZ is much lower than that of classic zirconia. However, the material strength of 5Y-PSZ (Table 1, 500–900 MPa according to Kwon et al. [7] and Čokić et al. [8]) exceeds by far the ISO 6872 threshold of 300 MPa needed for class 3 restorations such as single crown frames later provided with a partial or full veneering. Thus, the reduced strength of 5Y-PSZ should be no problem for anterior single crowns. Available laboratory studies indicate that overall mechanical properties are ranging between lithium disilicate-reinforced glass ceramics and conventional 3Y-TZP. An overview of novel zirconia materials and the change in material parameters depending on the yttria content was given by Zhang and Lawn [9]. The enhanced esthetics are further improved by the introduction of a color gradient. This especially enhanced its monolithic use in the anterior and premolar region of the mandible. However, especially in upper incisors a (facial) veneering still comes along with the possibility for harmonious and individual esthetics in contrast to the monolithic variant. A study on the fracture resistance of monolithic incisor crowns fabricated from 5Y-PSZ exceeded 1400 N for all tested specimens even after artificial aging, thus surpassing by far maximum possible bite forces [10]. A further study found that single crowns made from 5Y-PSZ of 0.8 mm uniform thickness benefit from using adhesive composite cements with regard to fracture resistance [11]. However, adhesive attachment of those restorations is a disadvantage as the luting process is time-consuming and technique sensitive [12]. Further, in contrast to glass ceramic attached bond strengths of zirconia are lower compared to etchable glass ceramic materials. Away from laboratory studies, clinical performance of monolithic anterior lithium disilicate or zirconia crowns was reported to be excellent. After up to 7.5 years of clinical service only 1% of the anterior monolithic crowns failed, whereas the number almost doubled for veneered restorations [13]. While monolithic zirconia crowns provide better survival rates, best esthetics are reached using ceramics veneering. For a dental technician it is, in general, not possible to ideally match the appearance of neighboring teeth with monolithic zirconia crowns. Therefore, it is of interest to compare different veneering concepts for anterior crowns with 5Y-PSZ frameworks with regard to the resulting fracture resistance, i.e., fully veneered crowns and cutback crowns with a minimized veneering.

Development of many zirconia veneering ceramic systems was made for 3Y-TZP and predates the development of 5Y-PSZ. These veneering ceramics are—in general—also indicated for use with 5Y-PSZ (*α*_T_ ≈ 10.0 10^−6^K^−1^) which has a slightly reduced coefficient of thermal expansion with regard to 3Y-TZP (*α*_T_ ≈ 10.5 10^−6^K^−1^). Because of this and other factors, crowns' fracture resistance may differ when 5Y-PSZ frameworks are veneered with these veneering ceramic systems. Evidence about issue is missing.

The objective of this laboratory study, therefore, was to evaluate fracture resistance of anterior crowns fabricated in a fully veneered design with minimum frame thickness and a cutback design (facial veneer only), respectively, each veneered with three different ceramic materials after and without artificial aging (both 5Y-PSZ frameworks). The study hypotheses were that the framework design (1), the choice of veneering ceramics (2), and artificial aging (3) have no statistical effect on test forces correlating with first major crack formation as well as test forces correlating with fracture.

## 2. Material and Methods

### 2.1. Test Groups and Sample Fabrication

**Figure 1:**
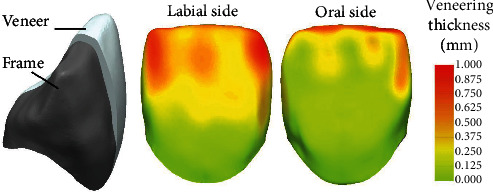
Left: cutback crown design with an anatomical frame (grey) reduced by an enamel veneering layer with up to 1.0 mm thickness at the incisal edge and the labial side (white, displayed only for half of the crown). Center: false color plot showing the veneering ceramic layer thickness on the labial side. Right: false color plot showing the veneering ceramic layer thickness on the oral side.

**Figure 2:**
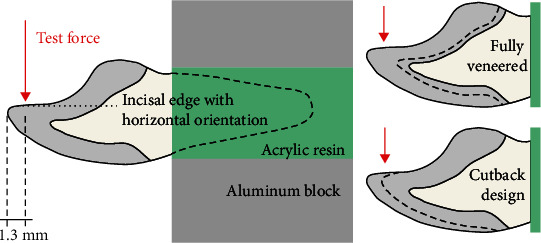
Left: test setup used for chewing simulation and fracture tests. The oral side of the incisal edge was oriented horizontally and mechanical loading took place at 1.3 mm distance from the incisal edge. Right: for both crown designs, the fully veneered, and the cutback variant, the dashed line indicates the interface area between zirconia frame and veneering ceramics.

Replicating a master die of a prepared tooth 11, CoCr dies (Remanium Star; Dentaurum, Ispringen, Germany) were casted. Based on a scan of the master die, an anatomical crown was designed with a complete wall thickness at the incisal edge of 2.3 mm. Two all-ceramic designs were finalized: (1) full veneering on a frame with constant thickness between 0.65 mm and 0.70 mm (fully veneered crown) and (2) partial reduction for the veneering at the labial surface by about 0.5–0.7 mm and at the incisal surface by 0.7–1.0 mm (cutback technique, Figure 1). For each of the two variants, 48 crowns were manufactured by one experienced dental technician of the dental laboratory of the Department of Prosthodontics, University of Heidelberg. Frameworks were milled (Cercon Brain Expert) from an extra translucent zirconia doted with 5 mol% Y_2_O_3_ (Cercon xt; Dentsply Sirona) and sintered (Cercon Heat Plus; Dentsply Sirona, Bensheim, Germany). In accordance with the manufacturer, the above mentioned framework thickness for the anatomically reduced framework was chosen slightly below the recommended minimum wall thickness of 0.7 mm, thus stating a rather critical case. Frameworks were veneered in two steps using layering technique and fired according to the manufacturer's instructions (Cergo Press; Dentsply Sirona, Bensheim, Germany). Silicon molds enabled standardized veneering of the crowns with rather identical final geometry. The crowns were finalized with two glaze firings. For veneering and glazing, three different veneering systems, i.e., (1) Cercon Ceram Kiss (CCK), (2) Ceramco PFZ (CPFZ), and (3) Celtra Ceram (CCer; Dentsply Sirona), were used. Information about all used ceramics can be found in Table 1. Thermal expansion of all veneering ceramics was between 9.0 10^−6^K^−1^ and 9.2 10^−6^K^−1^. The classic veneering materials CCK and CPFZ were leucite-free, whereas the recently developed CCer is a feldspathic ceramic containing leucite crystals. Thus, six test groups (*n* = 16) differing in two crown designs, and three veneering systems were compared in this investigation. In the following, each half of the crowns of each group was tested with regard to fracture resistance without or after an artificial aging process consisting of thermocycling followed by chewing simulation. All crowns were optically controlled for possible flaws or cracks using a light microscope (Stemi SR + Axiocam MRC; Zeiss, Oberkochen, Germany) prior to further processing. Before mechanical loading (i.e., after thermocycling for crowns subjected to artificial aging), crowns were adhesively attached to metal dies using MDP-containing composite cement (Panavia 21; Kuraray, Hattersheim, Germany). Prior to the luting process, metal dies were sandblasted (2 bar, 110 µm alumina) and inner surfaces of the crowns sandblasted (1 bar, 50 µm alumina), steam cleaned, dried, and conditioned using a MDP-containing ceramic primer (Clearfil Ceramic Primer; Kuraray). To enable later fixation for mechanical testing, the roots of the metal dies were embedded in metal molds with acrylic resin (Technovit 4071; Kulzer) such that the incisal edge was oriented horizontally (Figure 2).

### 2.2. Artificial Aging and Fracture Resistance Testing

Artificial aging comprised 10,000 thermocycles between 6.5°C and 60°C (Thermocycler TC01; SD Mechatronik, Feldkirchen, Germany) and 1,200,000 chewing cycles with a force magnitude of F_max_ = 86 N (Chewing Simulator CS4; SD Mechatronik, Feldkirchen, Germany). Vertical loads were applied on the horizontally oriented samples with a steel ball (6 mm in diameter) as antagonist at a distance of 1.3 mm from the incisal edge (Figure 1). During chewing simulation, samples were immersed in demineralized water. Completed crowns were again visually controlled for possible damages. Aged samples were stored in water for 30 days in total, including time durations spent for thermocycling and chewing simulation.

During fracture testing, samples were loaded in a universal testing device (Z005; Zwick/Roell) as described for chewing simulation with a steel indenter with spherical tip (6 mm in diameter) and a crosshead speed of 0.5 mm/min. Fracture was detected when the test force dropped to a level less than 25% of the maximum test force recorded for the respective sample. During fracture testing, body-borne sound signals were of interest as well. A steel needle placed on an acoustic piezo sensor contacted the crown surface, and sound signals were amplified and recorded with a sampling rate of 19.2 kHz (QuantumX MX840B and Catman Easy; HBK, Darmstadt, Germany).

Each fracture surface was observed with a digital microscope (Stemi SR + AxioCam; Zeiss, Jena) and assigned to one of the following four fracture modes: FM1, cohesive fracture within the veneer; FM2, cohesive fracture including the frame; FM3, cohesive fracture within the veneer and adhesive fracture along the frame/veneer interface; and FM4, cohesive fracture including the frame and adhesive fracture along the tooth/frame interface.

### 2.3. Evaluation of Test Data and Statistics

**Figure 3:**
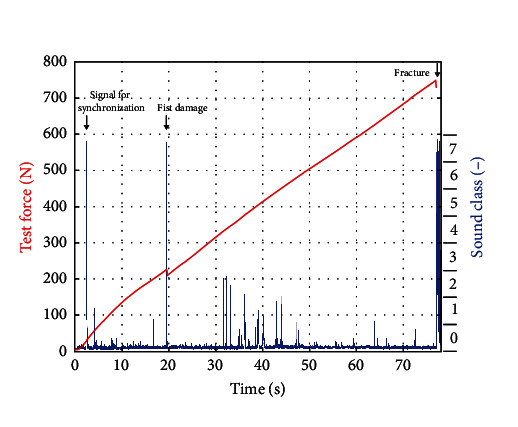
Time–force diagram for sound analysis during fracture tests. The example shows the recorded force (red line) and sound (blue line) signals during the fracture test of a cutback crown veneered with CCer after artificial aging. Depending on the sound magnitude, single events were assigned to seven sound classes (class 0 was associated with noise). A first damage of a crown was given when a drop in force coincided with a sound event of class 6 or 7.

Fracture resistance (ultimate force, F_u_) was associated with the maximum force during fracture testing. Crack growth within the ceramics, however, will start at lower force levels. The force corresponding with a first major damage (F_1d_) was identified by a drop in test force in combination with a sound magnitude exceeding 75% of the maximum recorded magnitude (Figure 3). An impulse given at the beginning of each fracture test served to synchronize recorded sound and force signals. In the case of visible cracks within the ceramics before fracture testing, the force at first damage was set to F_1d_ = 0 N if the crack occurred after thermocycling and to F_1d_ = 86 N if the crack was registered after chewing simulation. If a complete fracture happened during chewing simulation F_1d_ = *F*_u_ = 86 N was used for statistical evaluation of the respective sample. According to a mathematical model based on anatomical muscle data (physiological cross section, orientation) by Koolstra et al. [14], maximum possible single bite forces acting on any one tooth in all possible spatial direction were calculated for isometric and static clenching. For a protrusive horizontal force acting on an upper central incisor, it was calculated that the maximal static force a (typical) patient could possibly generate if all muscle contributions added up optimally was ≈100 N. Considering interindividual aspects and the fact that dynamic bite forces may exceed static ones, we defined a 200 N threshold for clinical recommendation for our *in vitro* test setup.

All statistical analyses were performed using (SPSS Ver. 28; IBM, New York, USA) at a significance level *α* = .05. Fracture resistances were visualized using a box-plot in addition to means (SD) as well as min/max. Nonparametric ANOVAs [15] based on sample ranks and Tukey post hoc tests were performed each for sample damaging and sample fracture to determine possible effects of (1) framework design, (2) veneering ceramics, and (3) artificial aging. Ranks for first damage and for fracture of the crowns increased according to the point in time at which damage/fracture was recorded during artificial aging. For crowns showing no damage/no fracture after aging, ranks increased further according to the respective F_1d_ and *F*_u_ values.

## 3. Results

### 3.1. Aging and Fracture Tests

**Figure 4:**
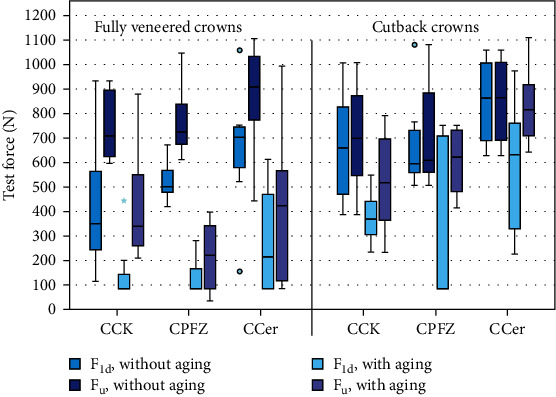
Boxplot diagram (median and quartiles) showing all results of the fracture tests.

All results of artificial aging and fracture tests are summarized in Table 2 and displayed in Figure 4. Fully veneered crowns were much more prone to cracks within the veneering (first damage) or chipping (fracture) during chewing simulation than crowns with cutback design. Only cutback crowns veneered with CCK and CCer were defect free after aging. First failures happened after 400,000 to 500,000 chewing cycles whereas visible cracks could be identified in some cases after a short aging time, in particular for crowns fully veneered with CPFZ.

All results of artificial aging and fracture tests are summarized in Table 2 and displayed in Figure 4. In general, mean forces associated with a first damage (F_1d_) and fracture resistances (F_u_) were significantly higher for veneered crowns with a cutback framework compared to crowns with an anatomically reduced a framework (*p* < 0.05). Only for the fracture resistance of unaged crowns, the factor “design” had no significant effect (Table 3). Artificial aging and selection of veneering ceramics strongly affected fracture behavior (Table 3). Aging significantly reduced fracture resistance. For all test groups with fully veneered crowns, fracture resistance dropped by at least 40% due to artificial aging. In contrast, the aging-induced decrease in mean fracture resistance lay between 15% and 26% for all crown with cutback design. Whereas many fully veneered crowns had cracks (first damage) in the veneering after chewing simulation, cutback crowns of groups CCK and CCer did not show such a behavior. When analyzing the effect of the different veneering ceramics, ranks for CCer associated with both, F_1d_ and F_u_, were significantly higher (*p* ≤ 0.007 for all tests) compared to the ranks found with CCK or CPFZ (Table 3). Of all test groups, only cutback crowns veneered with either CCK or CCer showed minimum F_1d_ values above this 200 N threshold.

### 3.2. Failure Modes

Examples of the different FM— if available for either fully veneered or cutback crowns are displayed in Table 4. Fully veneered crowns never failed according to FM2. Before aging, modes FM3 and FM4 were dominant. After aging almost half of the fully veneered crowns showed cohesive failure within the veneering ceramics (FM1). In contrast, no such behavior was found for the cutback crowns: About half of the aged and nonaged crowns failed according to FM1 or FM4, only some crowns according to FM2. In general, measured fracture forces increased with increasing FM number.

## 4. Discussion

The study hypotheses 1, 2, and 3 had to be rejected. The type of veneering, the choice of veneering material, and artificial aging each had a significant effect on fracture resistance.

The fact that zirconia crowns with a facial veneer yielded higher fracture loads values than the fully-veneered variant is in accordance with previous literature [16]. One explanation is that the zirconia frameworks come along with a substantially higher flexural strength compared to the veneering ceramics. In the recent study fracture testing was performed loading the unveneered (cutback variant) palatal aspect of the samples. In clinical studies—in analogy—fracture of the veneering/chipping is reduced or avoided, respectively in partially veneered restorations [17, 18]. The effect of aging as seen in the recent study was also theme of previous investigations. A reduction of loads needed to fracture restorations is associated with aging phenomena in the ceramic materials as initial flexural strengths are lowered with aging [19–21].

For all test groups without aging, crowns showed minimum fracture resistances ranging between about 400 N and 600 N. Consequently, directly after fabrication even the weakest crowns surpassed the threshold for clinical recommendation by far. Due to aging, measured fracture forces decreased and minimum values dropped below the threshold for all but two test groups (cutback crowns veneered with either CCK of CCer) remaining above 200 N. In this context one should discuss the artificial aging and fracture testing approach. Chewing simulation with a cycling loading of 86 N is rather high simulating force magnitudes of incisors. One-million and two-hundred-thousand cycles can be expected to simulate at least a clinical service of a restoration of 10 years (in 10 years, the equivalent of 328 such high cyclic *in vitro* loads on the incisor crown per day would be necessary to have a similar damaging effect)—if patients do not suffer from parafunctions. However, a reduction of fracture resistances along chewing simulation is not unexpected and multiply seen in previous studies [16, 19, 20]. The effect can be physically explained: ceramics, in particular veneering ceramics, suffer from subcritical crack growth during chewing simulation, which leads to a considerable reduction in bending strength (Belli et al. 2014). From our results it can be seen that mean forces correlated with a first damage (F_1d_) were drastically reduced by aging to about 25% (CPFZ)–42% (CCer) of their initial value for fully veneered crowns and 48% (CPFZ)–68% (CCer) for cutback crowns. In addition, water storage can lead to or speedup transformation of the metastable tetragonal phase into the monoclinic phase at low temperatures, so-called low temperature degradation [22]. However, such effects were of minor importance for this investigation.

As our study resulted in fracture resistances of crowns without or surviving chewing simulation of 412–874 N, previous studies observed mean values higher than 1000 N [16, 23]. However, the 5Y-PSZ restorations were monolithically designed and the study of Rosentritt et al. [23] used a rather uncritical test setup where a rubber sheet was placed between the samples and the testing pistil. The recently chosen test scenario is rather critical (oral application of force to the incisal edge). The recently observed results are—to the estimation of the authors—more clinically relevant since shearing forces can occur in the anterior region. In addition, another study which investigated 5Y-PSZ material [24] found mean strengths exceeding 500 N after different aging regimes. This strentgh is sufficient for class 4 according to ISO 6872.

In addition, Lawson et al. [11] found out that the selection of adhesive cement for luting 5Y-PSZ resulted in higher fracture resistance, however, not mandatory. In our study, glasionomer cement was used. To this end, in view of senseful applicability of the extra translucent 5Y-PSZ, one might weight if (full) veneering is needed in each case.

## 5. Conclusions

Within the limitations of this laboratory study, 5Y-PSZ based anterior crowns can be a viable treatment option. Framework design, choice of the veneering ceramics, and artificial aging show relevant effects on the fracture resistances. Concerted veneering ceramics should be used and partially veneering of the zirconia frameworks should be favored over full veneers.

## Figures and Tables

**Table 1:** Material parameters and composition of the used ceramics.

Brand name	Abbreviation	CTE *α*_T_ (10^−6^K^−1^)	Flexural strength (MPa)	Material composition
Cercon xt	—	10.1	>750	Zirconia, doped with 5 mol% yttria
Cercon Ceram Kiss	CCK	9.2	121	Silicon dioxide, alumina, etc.
Ceramco PFZ	CPFZ	9.1	90	Leucite-free esthetic glass-ceramic
Celtra Ceram	CCer	9.0	108	Leucite-reinforced feldspathic ceramic

**Table 2:** Forces recorded at first damage (*F*_1d_) and at fracture (*F*_u_) for all tested crowns. The number damaged crowns (*n*_1d,cs_) or fractured crowns (*n*_u,cs_) after chewing simulation. In case of such an event the respective test force was defined as the force magnitude used during chewing simulation (F_1d_ = 86 N, *F*_u_ = 86 N).

			First damage					Fracture				
			*n*_1d (cs)_ (−)	F_1d_ (N)				*n*_u,cs_ (−)	F_u_ (N)			
				Mean	SD	Min	Max		Mean	SD	Min	Max
Without aging	Fully veneered crowns	CCK	—	422	266	117	934	—	749	142	597	934
		CPFZ	—	525	81	421	675	—	767	144	612	1047
		CCer	—	660	254	157	1059	—	874	214	444	1104
	Cutback crowns	CCK	—	664	221	388	1007	—	706	216	388	1007
		CPFZ	—	671	185	508	1080	—	712	220	508	1080
		CCer	—	851	168	629	1059	—	851	168	629	1059

With aging	Fully veneered crowns	CCK	6	145	128	86	445	0	424	227	211	878
		CPFZ	6	131	83	86	282	3	223	134	86	398
		CCer	3	280	228	86	614	2	412	309	86	993
	Cutback crowns	CCK	0	378	103	235	550	0	524	201	235	792
		CPFZ	5	324	330	86	751	0	606	134	416	751
		CCer	0	581	267	227	974	0	830	155	643	1110

**Table 3:** Results of the ANOVAs for the force at first damage *F*_1d_ as well as the fracture resistance *F*_u_.

Factor	Force at first damage F_1d_		Fracture resistance F_u_	
	F	*p*	F	*p*
Design	27.033	**<0.001**	7.668	**0.007**
Veneering	8.914	**<0.001**	7.243	**0.001**
Aging	62.643	**<0.001**	42.255	**<0.001**
Design*∗* veneering	0.158	0.854	1.438	0.243
Design*∗* aging	0.858	0.357	16.208	**<0.001**
Veneering*∗* aging	0.328	0.721	0.197	0.822
Design*∗*veneering*∗* aging	0.304	0.739	1.353	0.264

*Note:* Significant values are highlighted using bold font.

**Table 4:** Observed fracture modes. With increasing fracture mode number, an overall increased fracture resistance was observed within each test group. Exemplary pictures of fractured crowns are displayed. Differences in fracture surface roughness are due to the different structure of the veneering ceramics. CCer samples showed rough fracture surfaces in the veneering due to the leucite crystals whereas CCK und CPFZ samples had rather smooth fracture surfaces.

	Fracture mode			
	FM1	FM2	FM3	FM4
Fully veneered crowns	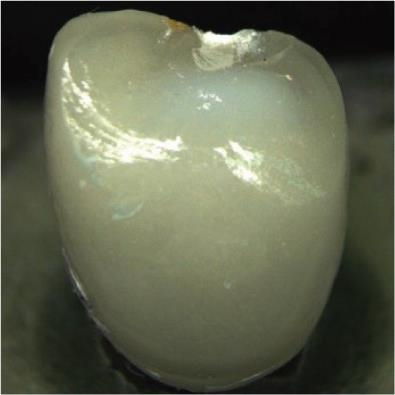	x	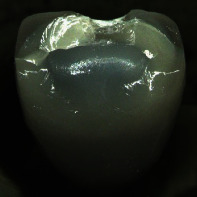	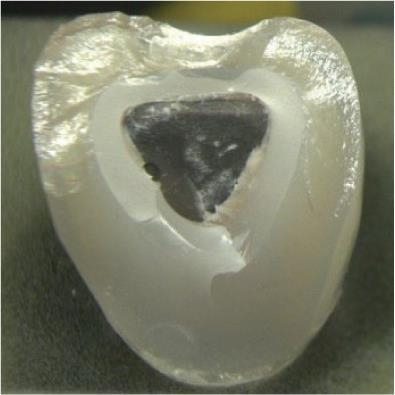

Cutback crowns	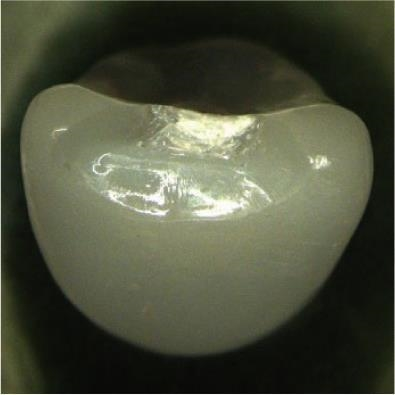	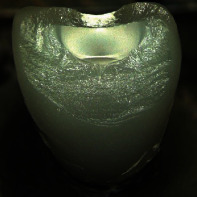	x	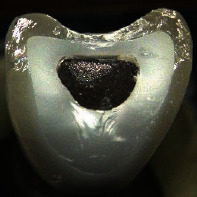

## Data Availability

Original source data can be made available on request by the corresponding author.
